# Halo-free quantitative phase imaging via physics-constrained self-supervised network

**DOI:** 10.1117/1.JBO.31.7.076503

**Published:** 2026-07-07

**Authors:** Haobin Ye, Tianhe Wang, Lin Liu, Liangwei Li, Xiaohui Du, Ruqian Hao, Juanxiu Liu, Jing Zhang

**Affiliations:** University of Electronic Science and Technology of China, School of Optoelectronic Science and Engineering, Chengdu, China

**Keywords:** self-supervised learning, quantitative phase imaging, halo effect, physics-constrained neural networks

## Abstract

**Significance:**

The halo effect arising from limited spatial coherence and system noise severely compromises the accuracy of white-light diffraction phase microscopy, hindering its broader application in high-fidelity label-free biological observations.

**Aim:**

We propose and evaluate a physics-constrained halo-free self-supervised network (PC-HFSSN) designed to suppress the halo effect in partially coherent imaging systems without requiring experimentally inaccessible ground-truth phase labels.

**Approach:**

Our approach embeds a differentiable forward optical model directly into the network’s loss function. By integrating the physical spatial smoothness constraints of halo fields with a Gaussian illumination approximation, our lightweight architecture was trained on multi-scale synthetic data to accurately isolate and remove halo fields from the acquired images.

**Results:**

Trained on diverse sample data, PC-HFSSN demonstrated robust “simulation-to-reality” generalization. Experimental validation of both simulations and dynamic live cells confirmed that the proposed method effectively eliminated the halo effect while preserving high-frequency phase accuracy and maintaining high computational efficiency.

**Conclusions:**

PC-HFSSN provides a robust, physically interpretable, and self-supervised solution for high-throughput quantitative phase imaging by balancing high fidelity and processing speed without the need for paired training data.

## 1 Introduction

Quantitative phase imaging (QPI) enables label-free, high-contrast visualization and precise quantification of transparent specimens, including living cells and microstructured materials. By retrieving the phase delay induced by the optical path length of the sample, QPI yields critical biophysical parameters, such as dry mass, refractive index, and morphological dynamics, which are essential for long-term biological monitoring and surface metrology.^1^^,^^2^ Among QPI modalities, white-light diffraction phase microscopy (wDPM)^3^ is notable for its common-path interferometric stability and single-shot acquisition capability. Typically implemented via 4f systems or diffraction gratings, wDPM facilitates the real-time recording of dynamic cellular processes with high temporal resolution.^4^^,^^5^

**Fig. 1:**
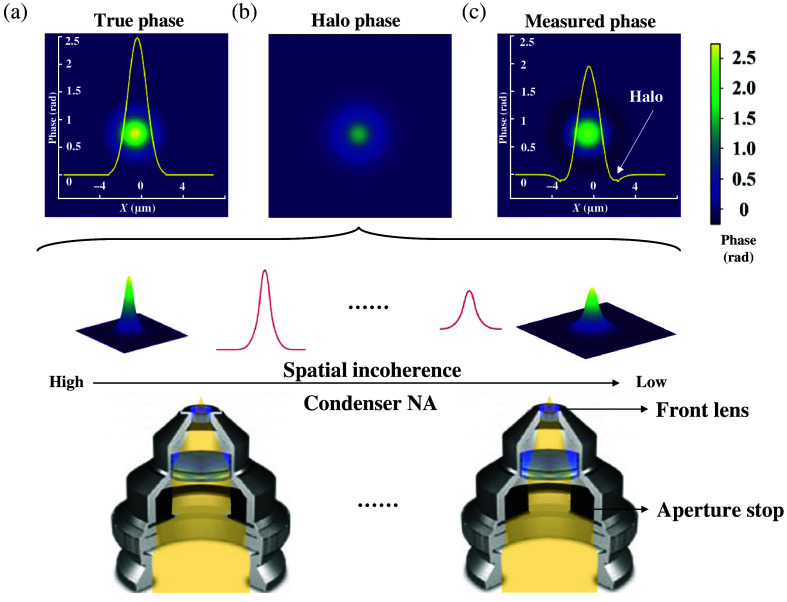
Halo effect formation. (a) Simulation of the true phase for a microsphere sample. (b) Simulation of the halo phase under ideal light-field conditions. (c) Simulation of the measured phase with a halo, which is the difference between panels (a) and (b). A low condenser NA enhances the spatial coherence of the illumination field. A high NA diminishes the spatial coherence of the illumination field. Owing to low spatial coherence, the interference fringe contrast is reduced, and the spectrum broadens, resulting in a non-uniform bias field that appears as a blurred halo around the sample.

Despite these advantages, wDPM operated with broadband extended sources, such as halogen lamps or light emitting diodes, suffers from a fundamental trade-off between photon throughput and spatial coherence. Partially spatially coherent illumination improves photon efficiency and practical imaging speed; however, it also weakens the mutual interference of low-spatial-frequency components across the field of view, resulting in a halo effect. As illustrated in Fig. 1, the halo effect appears as bright or dark pseudo-contours around the object boundaries and as a depression or bias in the phase values in slowly varying regions. Although the dynamic range may remain partially preserved, this artifact introduces systematic quantitative errors and can obscure low-contrast cellular structures, thereby compromising dry mass estimation, refractive index analysis, and morphological interpretations.

The physical origin of the halo effect is closely related to the coherence transfer properties of partially coherent optical systems. The general theoretical description of partially coherent imaging is provided by the transfer cross-coefficient (TCC) formalism proposed in the Hopkins framework,^6^^,^^7^ in which the recorded field or intensity response is determined by the coupling relationship among object spatial frequency pairs, the illumination source, and the imaging aperture, which leads to a self-TCC description of image intensity. However, in wDPM, the phase distribution is obtained through off-axis interference between the sample field and the spatially filtered reference field. Therefore, a strictly relevant direct description is the cross-TCC between the imaging and reference beams. In the partially spatially coherent model proposed by Edwards et al.,^8^ limited condenser spatial coherence and non-ideal reference beam filtering produce a quasi-high-pass phase response, resulting in the attenuation of low-spatial-frequency phase components, reduced central phase, and an edge halo effect.

Halo correction has also been studied in the context of the transport of intensity equation (TIE). TIE-based methods recover the phase from the axial intensity variation by associating the longitudinal intensity derivative with the transverse phase gradient. The regularized single-distance method proposed by Paganin et al.^9^ demonstrated that physics-based prior information can stabilize the phase retrieval process and suppress low-frequency artifacts during propagation. However, the TIE is typically suitable only for out-of-focus or propagation-based intensity contrasts, whereas wDPM recovers the phase from common-path off-axis interferograms. Therefore, halo correction in wDPM requires a forward model that can account for the effects of image reference interference and reference beam filtering under coherence constraints.

Recent research on halo-free imaging has primarily focused on phase contrast and diffraction phase imaging. Nguyen et al.^10^ demonstrated a physics-based halo removal strategy for phase-contrast microscopy by modeling the partially coherent imaging process and reconstructing halo-free phase information from multiple intensity measurements. This study established the feasibility of physics-guided halo suppression; however, the requirement for additional measurements or iterative computations can restrict real-time applications. Kandel et al.^11^ further developed real-time halo correction strategies for phase contrast imaging. In wDPM and related QPI systems, Nguyen et al.^12^ analyzed how condenser spatial coherence and Fourier plane reference filtering determine the high-pass phase response. However, traditional strategies employed in these studies to mitigate the halo effect have inherent limitations. Reducing the illumination aperture improves coherence but drastically attenuates the photon flux, reducing the signal-to-noise ratio and compromising the temporal resolution in live-cell imaging.^13^ Computational approaches, such as Hilbert-transform-based methods, offer efficiency but assume narrowband signals, often leading to over-smoothing of complex phase gradients and loss of structural details. Alternatively, regularized iterative algorithms provide higher reconstruction accuracy but require computationally expensive optimization loops in the complex domain, rendering them impractical for real-time applications.^14^

Recent developments have shown that learned reconstructions can be interpreted as extensions of classical regularized inverse problems,^15^^,^^16^ in which the network is constrained by a known forward operator, noise model, and prior information. Deep learning has emerged as a promising approach for halo suppression. Supervised models, such as halo-free deep neural networks (HFDNNs), have demonstrated effective denoising capabilities.^17^ However, their deployment is hindered by the ground truth paradox in QPI, in which acquiring perfectly paired, artifact-free phase labels for real biological samples is experimentally infeasible.^18^^,^^19^ Consequently, supervised models trained on limited or imperfect datasets often fail to generalize across diverse sample morphologies and refractive index distributions. Although self-supervised learning^20^^,^^21^ has transformed inverse problem-solving^22^^,^^23^ in modalities such as computer tomography (CT) and magnetic resonance imaging (MRI) by leveraging unlabeled data,^24^^–^^28^ its application to digital holographic QPI remains largely unexplored. Specifically, current frameworks lack the integration of interferometric physical priors, such as the optical transfer function (OTF) and phase continuity, into the network design, thereby limiting their interpretability and robustness under low-coherence illumination.

To address these limitations, we propose a physics-constrained halo-free self-supervised network (PC-HFSSN) for halo correction in wDPM. PC-HFSSN does not treat halo removal as generic image enhancement. Instead, it embeds the process of wDPM halo effect formation into physics-constrained self-supervised network training. Specifically, the experimentally inaccessible components of the illumination function and spatial light modulator response were approximated using illumination model fitting and angular spectrum diffraction theory.^29^ Therefore, the method avoids the need for experimentally paired halo-free labels while retaining explicit physical constraints through the forward model and phase priors. The resulting model captures the dominant low-frequency coherence bias responsible for halo formation while remaining suitable for end-to-end self-supervised optimization. The network was implemented with a lightweight U-Net architecture and trained using synthetic phase distributions of multi-scale structures, including microspheres and human red blood cells (RBCs), without requiring an experimentally measured halo-free ground truth.

**Fig. 2:**
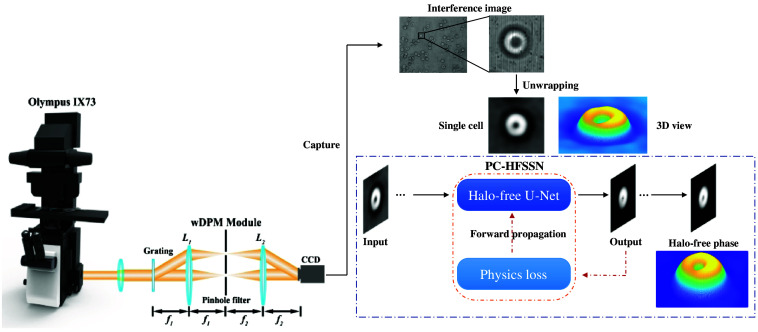
Framework of self-supervised phase retrieval in quantitative phase imaging.

The main contributions of this study are threefold. First, we established an interpretable self-supervised halo-removal framework that links deep reconstruction to a physically motivated optical forward model (Fig. 2), thereby reducing the dependence on paired experimental labels. Second, by combining synthetic phase priors with a differentiable coherence-aware degradation model, PC-HFSSN enables simulation-to-reality generalization for live-cell wDPM data while preserving spatial details. Third, the proposed method achieves a reconstruction accuracy comparable to that of rigorous iterative algorithms while improving computational efficiency to support fast halo-free QPI. Therefore, this study provides a practical bridge between classical partially coherent imaging theory and physics-informed deep learning and offers a generalizable solution for halo suppression in low-coherence interferometric microscopy. A preliminary version of this work was presented in conference form at the 17th International Conference on Signal Processing Systems (ICSPS).^30^ The present article substantially extends that work by providing a more complete wDPM halo-effect model, expanded theoretical analysis, additional experimental validation, and more comprehensive quantitative evaluation.

## 2 Proposed Method

### 2.1 Optical System

**Fig. 3:**
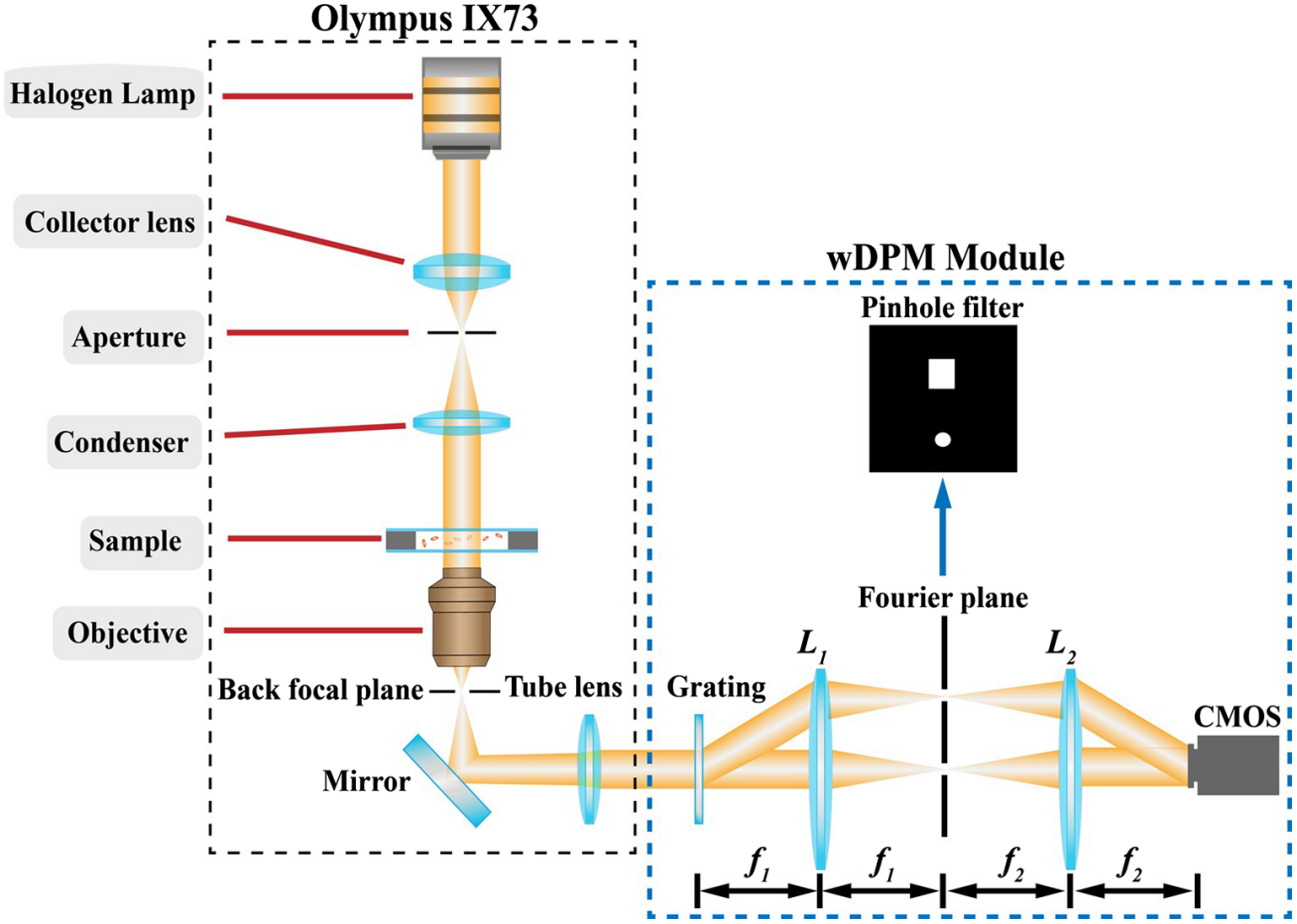
Schematic of the white-light diffraction phase imaging system.

The white-light diffraction phase microscopy system integrates a halogen-lamp-illuminated inverted microscope with a phase plane diffraction grating, 4f lens system, spatial filter, and camera to achieve common-path interferometry. In this configuration, the incident light field is first diffracted into multiple orders by the grating, where the 0th-order (reference) and +1st-order (object) beams are isolated through aperture screening and spatially separated via a vacancy design in the Fourier plane filter. These beams subsequently interfere at the camera plane after passing through a Fourier transform lens, generating single-frame holograms that encode quantitative phase information. By leveraging the sub-micron-scale coherence length of white-light illumination, the system inherently suppresses laser speckle noise and enhances aperture sensitivity, thereby enabling high-contrast imaging of transparent specimens under ambient light. However, the limited spatial coherence area of the extended light source, which is significantly smaller than the measurement field of view, induces a structure-dependent halo effect in the reconstructed phase images. These artifacts manifest as low-frequency distortions at the sample edges and central contrast reduction, fundamentally arising from incomplete interference between the object and reference beams. This physical limitation necessitates integrated suppression strategies that combine forward physical modeling and computational corrections to ensure accurate phase retrieval (Fig. 3).

The light source for the microscope was spatially coherent white light obtained from a halogen lamp commonly used in commercial microscopes, with a central wavelength of 574 nm and a bandwidth of 170 nm. According to the equation for calculating the coherence length $l_{c}=\lambda^{2}/\Delta \lambda$, known that the coherence length of white light is 1.9  μm, and the numerical aperture (NA) of the light source is related to its spatial coherence, which can be calculated by ${\mathrm{NA}}_{c}=\lambda /\pi l_{c}$ known value to obtain the NA value of 0.096. The microscope was equipped with a 40× magnification, 0.65-NA plan achromatic objective lens. To ensure that all diffraction orders fully contain all sample information, according to the Nyquist sampling theorem, the grating constant of the diffraction grating must be less than or equal to half the diameter of the smallest diffraction spot on the imaging plane. Furthermore, as the white-light diffraction phase microscopy system employs a fully off-axis digital holographic configuration, a large angle between the object light and reference light is required to avoid aliasing errors in the Fourier spectrum. Therefore, a diffraction grating with a grating constant of 10  μm was placed on the microscope imaging plane for spectral separation. For a 4f imaging system, to ensure that the CMOS camera can correctly sample the off-axis interference image, according to the Nyquist sampling theorem, the sampling frequency of the camera must be greater than twice the maximum frequency of the digital holographic image, and the maximum frequency of the camera is also related to its pixel size. The pixel size of the camera used was 2.5  μm. In addition to meeting the magnification requirements defined by the pixel size, the imaging system must ensure that the 0th-order and +1st-order light waves generated by the diffraction grating can pass through completely and reach the CMOS camera imaging plane. Therefore, an output Fourier plane pinhole as a spatial light modulator (SLM) capable of low-pass filtering must be introduced. Specifically, in a white-light diffraction phase imaging system, the center of the spatial filter is a circular aperture with a diameter of 200  μm, used to perform low-pass filtering on the 0th-order light after diffraction by the grating. The position of the +1st-order spectrum center is 8.61 mm from the center of the circular aperture, which can be calculated using the diffraction grating equation,

$$\Delta L=f_{1}\lambda /\Lambda ,$$ (1)

where ΔL is the distance between the 0th-order spectrum and +1st-order spectra. To ensure that the +1-order spectrum can pass through completely and that diffraction light waves of other orders are completely blocked, the filter aperture size for the +1-order spectrum was designed as a 10  mm×5  mm rectangular aperture, while the entire spatial filter was a 100  mm×50  mm opaque metal mask plate.

The interference pattern formed by the superposition of the object and reference lights was recorded as intensity fringes by the camera, wherein the detected signal represented the time-averaged intensity distribution over the integration period of the camera. The measured light intensity (or irradiance distribution) can be expressed as

I(x,y)=I1+I2+2 Re⟨U1(t)U2*(t)⟩t, (2)

where $I_{1}$ and $I_{2}$ are the light intensity of the 0th-order and the +1-order; both light intensities exhibit a correlation function $\langle U_{1}(t)U_{2}^{*}(t)\rangle_{t}$, which is the cross-correlation function of the light fields.

### 2.2 Halo Effect

In off-axis holographic systems, both the object and reference beams traverse identical optical paths and components, resulting in a zero-time delay among them. Consequently, the mutual interference function of the light field can be expressed as

$$\Gamma_{s,r}(r,0)={\left\langle U_{s}\left(r,t\right)U_{r}^{*}\left(r,t\right)\right\rangle }_{t}.$$ (3)

According to the generalized Wiener–Khinchin theorem, for a wide-sense stationary random process, the power spectral density in the frequency domain is the Fourier transform of the autocorrelation function in the time domain. This fundamental relationship is established by defining the autocorrelation function as the statistical average (or, equivalently, the time average for ergodic processes) over time. Consequently, the theorem provides a rigorous time-domain–frequency-domain conversion framework, linking the temporal statistics of a signal to its spectral energy distribution, so $\Gamma_{s,r}(r,0)$ can be expressed as

$$\Gamma_{s,r}(r,0)=\int W_{s,r}(r,w)dw=\int U_{s}(r,w)U_{r}^{*}(r,w)dw.$$ (4)

In the wDPM system, spatial positive first-order light can pass completely through the beam splitter; thus, the complex amplitude of the object light field can be expressed as

$$U_{s}(r,w)=T(r)U_{i}(r,w),$$ (5)

where T(r) is the transmittance function of the sample, and $U_{i}(r,w)$ is the illumination light field of the sample. Similarly, the 0-level light was used as the reference light after filtering, which can be expressed as

$$U_{r}(r,w)=[T(r)U_{i}(r,w)]⊗h_{0}(r),$$ (6)

where ${⊗}_{r}$ represents the spatial convolution, and $h_{0}(r)$ corresponds to the Fourier transform of the 0-level filtering spatial transmittance function. Substituting the above expression into the original equation yields the following simplified expression for the light-field mutual interference function,

$$\Gamma_{s,r}(r,0)=\int {\left\langle T(r)U_{i}(r,w){\left[[T\left(r^{\prime }\right)U_{i}(r^{\prime },w)]⊗h_{0}(\Delta r)\right]}^{*}\right\rangle }_{t}dw\;=T(r)\iint_{-\infty }^{\infty }\Gamma_{i}(\Delta r,0)\iint_{-\infty }^{\infty }W_{i}(r,r^{\prime },w)h_{0}^{*}(\Delta r)T^{*}(r^{\prime })d^{2}r^{\prime }\;=T(r){\left[T(r){⊗}_{r}h(r)\right]}^{*},$$ (7)

where $W_{i}(r,r\prime ,w)$ is the cross-spectral density function of the sample illumination light field, and the light source mutual coherence function $\Gamma_{i}(r,0)$ reflects the coherence of the illumination light field, and there exists $h(r)=\Gamma_{i}^{*}(r,0)h_{0}(r)$. Therefore, the phase ϕ(r) of the sample under the current system transmission function T(r) can be solved as follows:

$$\phi_{m}(r)=\phi_{t}(r)-\arg[T(r){⊗}_{r}h(r)]\;=\phi_{t}(r)-\arg[(\Gamma_{i}^{*}\Pi_{\mathrm{SLM}}){⊗}_{r}e^{i\phi_{t}\left(r\right)}],$$ (8)

where $\Pi_{\mathrm{SLM}}$ is the modulation function in the optical system. Therefore, there is a difference in the low-frequency components between the measured phase $\phi_{m}(r)$ and the true phase $\phi_{t}(r)$. This low-frequency component causes a reduction in the actual measured value and the symmetrically and evenly distributed phase negative value phenomenon, that is, the halo effect.

### 2.3 Forward Propagation Physical Model

The forward model used in PC-HFSSN is based on the mutual coherence equation of a partially coherent wDPM. In an ideal fully coherent interferometric system, the demodulated phase should match the object height phase. However, in a practical wDPM, the influence of extended white-light illumination, the finite numerical aperture of the condenser, and spatial filtering of the reference beam alter the mutual interference term between the sample and reference fields. Consequently, the recovered phase contains a low-frequency suppression component, which manifests as phase underestimation in slowly varying regions and as halo-like pseudo-contours near object boundaries. The filtering term associated with halos can be represented as the combined response of the illumination coherence function with the reference beam and SLM filtering functions. In the frequency domain, this effective response is expressed as

$$h_{i}(r)\approx \Gamma_{i}^{*}(r)\Pi_{\mathrm{SLM}}(r),$$ (9)

where $\Gamma_{i}(r)$ denotes the source-dependent illumination cross-correlation or mutual-coherence term, the superscript * denotes complex conjugation, and $\Pi_{\mathrm{SLM}}(r)$ represents the finite passband introduced by the SLM-based reference beam filtering. This expression summarizes the two physical origins of the halo term: the finite spatial coherence of the illumination and the non-ideal low-pass filtering of the reference field.

For an ideal uniform circular condenser aperture, $\Gamma_{i}$ can be related to a jinc-type mutual intensity function.^31^ However, in the actual illumination system, broadband white light from a halogen source is shaped by the illumination optics and the condenser aperture. Owing to finite source size, lens aberrations, diffraction at aperture boundaries, and weak optical scattering, the effective angular spectrum may deviate from an ideal pillbox, or circ, distribution and exhibit a smooth roll-off near the aperture edge. We therefore model the effective illumination source using a Gaussian-like profile constrained by the condenser numerical aperture, rather than an ideal top-hat function

$$\Gamma_{i}\approx F^{-1}(e^{-\frac{{\left\|k\right\|}^{2}}{{2\sigma }_{k}^{2}}}).$$ (10)

The reference filtering term is modeled as an NA-defined circular passband

$$\Pi_{\mathrm{SLM}}(k)=F^{-1}(\{\begin{array}{cc} 1, & \left\|k\right\|\leq k_{c}\\ 0, & \left\|k\right\|>k_{c} \end{array}).$$ (11)

The width and cutoff frequency are parameterized by optical quantities as

$$\sigma_{k}\approx \frac{2\pi \overset{¯}{n}{\mathrm{NA}}_{c}}{\lambda },\;k_{c}=\frac{2\pi \overset{¯}{n}{\mathrm{NA}}_{f}}{\lambda },$$ (12)

where λ is the illumination wavelength, n is the average refractive index of the medium, ${\mathrm{NA}}_{c}$ denotes the effective condenser-based source numerical aperture, and ${\mathrm{NA}}_{f}$ denotes the numerical aperture associated with the SLM-based reference filtering. In our wDPM system, the effective condenser-based source numerical aperture was set to 0.095, whereas the SLM-based reference filtering numerical aperture was 0.0072. These values were used to determine the Gaussian width and cutoff frequency. Given the true object phase $\phi_{t}(r)$, the complex transmittance of a weakly absorbing phase object is approximated as

$$\phi_{m}(r)=\phi_{t}(r)-\arg[h_{i}(f){⊗}_{r}e^{i\phi_{t}\left(r\right)}].$$ (13)

This expression indicates that the measured wDPM phase contains the object phase minus the smoothed, coherence-related phase components introduced by finite spatial coherence and non-ideal reference beam filtering. By simplifying the calculation of halo propagation to convolution operations, frequency-domain filtering, and accelerated Fourier transforms, a comprehensive simulation of diffraction, aberrations, and halo effect in practical optical systems was achieved.

### 2.4 Physics-Constrained Self-Supervised Network Structure

Conventional supervised methods require accurate ground-truth phase or refractive index (RI) distributions for training. However, the RI of biological samples changes dynamically owing to environmental factors and the cell cycle, making it experimentally challenging to obtain perfectly paired artifact-free annotations.^32^ Obtaining paired labels with halo-contaminated and halo-free phase maps is experimentally challenging in biological QPI systems. Our physics-constrained self-supervised learning paradigm explicitly incorporates a physical constraint module, in which supervision is provided by a differentiable wDPM forward model and physically motivated phase priors, and provides a distinct advantage for the continuous, high-throughput monitoring of dynamic live cells.

**Fig. 4:**
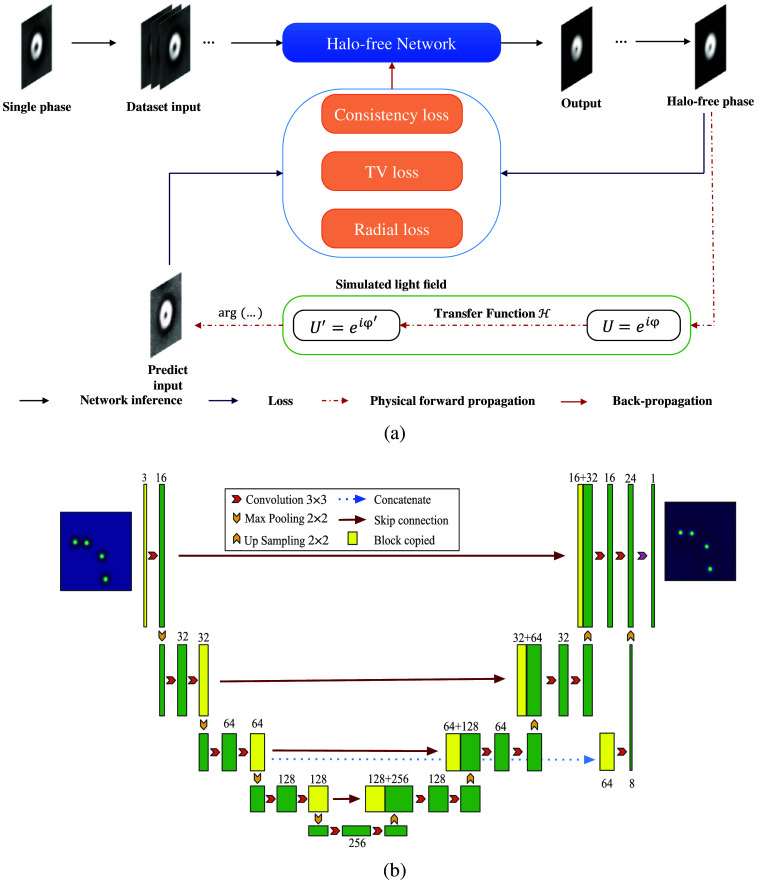
Schematic of the proposed PC-HFSSN architecture. (a) Pipeline. (b) Network.

To satisfy the specific requirements of quantitative phase retrieval while leveraging the proven efficacy of the U-Net architecture in biomedical imaging,^33^ we propose an optimized and lightweight U-Net backbone for our self-supervised framework (Fig. 4). Based on a symmetric encoder–decoder topology, the network employs multi-scale feature fusion and skip connections to achieve computationally efficient and accurate halo suppression results. In the encoder, an initial 3×3 convolution expands the single-channel input into three channels. This is followed by a double convolution block utilizing instance normalization (IN) and rectified linear unit (ReLU) activation to enhance initial feature extraction.^34^

The encoder comprises three downsampling levels, each containing a double convolution block with a doubled channel capacity, followed by a 2×2  max-pooling operation. This progressively compresses the spatial resolution to 1/8 of the original size and extracts the multi-scale contextual features of the phase distribution. Notably, we replaced the standard batch normalization with IN across all downsampling modules, as IN is more stable and better suited for small batch sizes typical in phase map training scenarios. At the bottleneck, a 128-channel convolution block processes the lowest-resolution feature map to capture global semantic information while mitigating the vanishing gradient problem.

In the decoder, transposed convolutions gradually restore the spatial resolution. To compensate for the spatial information lost during downsampling and improve edge localization, multi-scale features from the encoder are integrated via channel concatenation. Furthermore, we improved the standard skip connection strategy by introducing an intermediate feature extraction module. Conventional methods often rely on simple feature concatenation between the first and last layers, which can lead to the loss of fine structural details in complex phase distributions. To address this, we extracted 8-channel features from the second encoder layer (64 channels), upsampled them, and concatenated them with the 16-channel decoder output. This fused representation is then compressed through an 11-channel fusion layer to achieve robust cross-level feature complementarity. This customized cross-connection mechanism significantly enhances the model sensitivity to low-frequency morphological features typical of halo boundaries, providing superior generalization for dynamic biological samples compared with traditional architectures.

Based on the lightweight halo restoration U-Net skeleton, which is based on reducing the number of core channels, and the halo phase physical constraint model proposed in the previous section, a complete self-supervised halo restoration network architecture driven by differentiable optical propagation was constructed by embedding the optical system propagation process of angular spectrum diffraction in a differentiable layered form to establish a closed loop for forward and backward propagation. In forward propagation, the network simulates the optical imaging process through a diffraction propagation framework and reconstructs the halo-free phase by propagating the input phase distribution through multilevel angular spectrum diffraction propagation. Backpropagation was used to simulate the optical decoding mode of halo formation, and the predicted phase was backpropagated to the object plane to generate a halo phase distribution that approximates the actual optical system acquisition. The physical consistency loss is constructed by the mean square error between the input halo phase and the predicted halo phase, which can be expressed as

$$L_{1}=\frac{1}{M\times N}\overset{M}{\underset{i}{\sum }}\overset{N}{\underset{j}{\sum }}{\left\|\phi_{m}(i,j)-\phi_{\mathrm{pred}}(i,j)\right\|}_{2}^{2}.$$ (14)

To further ensure that the halo distribution has a realistic physical meaning and suppresses artifacts, the ReLU operator is applied only to halo amplitude values in the penalty branch to penalize physically unreasonable excess energy, rather than enforcing a non-negative sign phase. The reconstructed phase itself is allowed to take signed values, effectively avoiding the vanishing gradient problem. Simultaneously, an L1 regularization term is combined to impose sparse constraints on the halo values, penalizing non-zero parameters to prompt the model to focus on key halo regions and thereby suppress redundant artifact signals.^35^ Furthermore, a mask generated via a non-iterative transform was used to locate the critical halo regions. Within this region, gradient penalties are applied to force the predicted phase to exhibit sufficient gradient changes at the boundaries covered by the mask, thereby preventing the halo energy distribution from becoming excessively flat. Each point on the halo phase is guided to focus on the light source center region (satisfying the gradient rise condition). Radial attenuation weights were innovatively introduced to force the phase value to exponentially decay with increasing distance from the center to the background phase under non-negative constraints, satisfying the actual observation law of phase attenuation. This joint optimization strategy synergistically acts from both mathematical regularity and physical rationality dimensions, satisfying the non-negativity requirement of the halo intensity distribution while guiding energy toward the center through local gradient constraints, ultimately enhancing the physical consistency and visual quality of the reconstruction. The joint loss function can be expressed as

$$L_{2}={\left\|\mathrm{ReLU}\{\arg[h_{i}(f){⊗}_{r}e^{i\phi_{t}(r)}]\}\right\|}_{1},$$ (15)

$$L_{3}=E_{M}[\mathrm{ReLU}\{\epsilon -\|\nabla \phi_{t}\|\}],$$ (16)

$$\text{Loss}=L_{1}+\lambda_{1}L_{2}+\lambda_{2}L_{3}.$$ (17)

The network is optimized using an adaptive learning rate scheduling, with an initial learning rate of ${1\times 10}^{-3}$, a reduction factor of 0.5, and a minimum learning rate of ${1\times 10}^{-6}$; the network output is constrained to comply with wDPM system principles, and the network is driven to learn features consistent with the halo effect, thereby enabling the physical consistency loss to be backpropagated through the complete degradation model.

## 3 Experiment Design

### 3.1 Benchmark Algorithm Setup

For the phase distribution of the real samples, we could only obtain a reasonable range of the true value of the halo-free phase. To compare the self-supervised network more accurately, we conducted benchmark experiments on the halo phase based on traditional methods, including the traditional non-iterative Hilbert transform algorithm, which can remove the edge artifacts of the halo phase alone, and the iterative deconvolution optimization algorithm to obtain the comparison results for eliminating the halo phase.

The non-iterative Hilbert halo phase recovery algorithm directly separates and suppresses artifacts from the phase image containing the halo by combining frequency-domain directional filtering with the Hilbert transform.^36^ First, the input phase image is represented by a two-dimensional Fourier transform to obtain the frequency-domain representation, and the frequency domain is then modulated by a direction-sensitive filter group, wherein Lc is the spatial cutoff frequency of the halo feature, and the direction angle is preset (typically 0, 60, and 120 deg to cover the anisotropic halo). The filter design follows the frequency-domain characteristics of the Hilbert transform: a 90-deg phase shift is applied to low-frequency components (halo-dominated areas) below (positive and negative frequencies are multiplied by), whereas high-frequency details maintain the original phase to preserve the real structure. The imaginary part of the filtered spectrum is obtained following an inverse Fourier transform to generate Hilbert response images in multiple directions, the physical meaning of which is the orthogonal phase field of the halo in different directions. Finally, the results are fused by taking the maximum value operation pixel by pixel: the halo-free area retains the original phase (because the high frequency is not disturbed), the halo area suppresses artifacts through a multi-directional response, and the maximum selection strategy is combined to avoid single-direction deviation.

The iterative deconvolution optimization algorithm relies on the halo effect equation mentioned in Sec. 2 to construct the total variation term and uses the L2 norm to minimize the difference between the measured and reconstructed phases.^37^ Simultaneously, combined with total variation (TV) regularization,^38^ the gradient sparsity assumption is used to suppress random noise, and a weight parameter is introduced to balance data fidelity and regularization strength. The specific implementation of its optimization mainly uses the limited-memory Broyden–Fletcher–Goldfarb–Shanno (BFGS) algorithm with box constraints (L-BFGS-B) to solve the halo optimization problem under non-negative constraints.^39^ The optimization objective function is expressed as

$$\phi^{†}=\arg\;\min\{{\left\|\phi_{m}(r)-\phi (r)+\arg[h_{i}(f){⊗}_{r}e^{i\phi_{t}(r)}]\right\|}_{2}^{2}+\lambda \mathrm{TV}(\phi )\},$$ (18)

$$\mathrm{TV}(\phi )=\int d^{2}r\sqrt{\nabla_{x}^{2}(r)+\nabla_{y}^{2}(r)}.$$ (19)

Owing to the introduction of the convolution phase term containing spatial convolution and phase extraction, and the L-BFGS-B algorithm requires that the optimized objective function and the corresponding gradient be real functions, the gradient of the data fidelity term is calculated by combining complex conjugate gradient optimization, and the gradient of the TV regularization term is calculated by divergence.

### 3.2 Simulation Setup

**Fig. 5:**
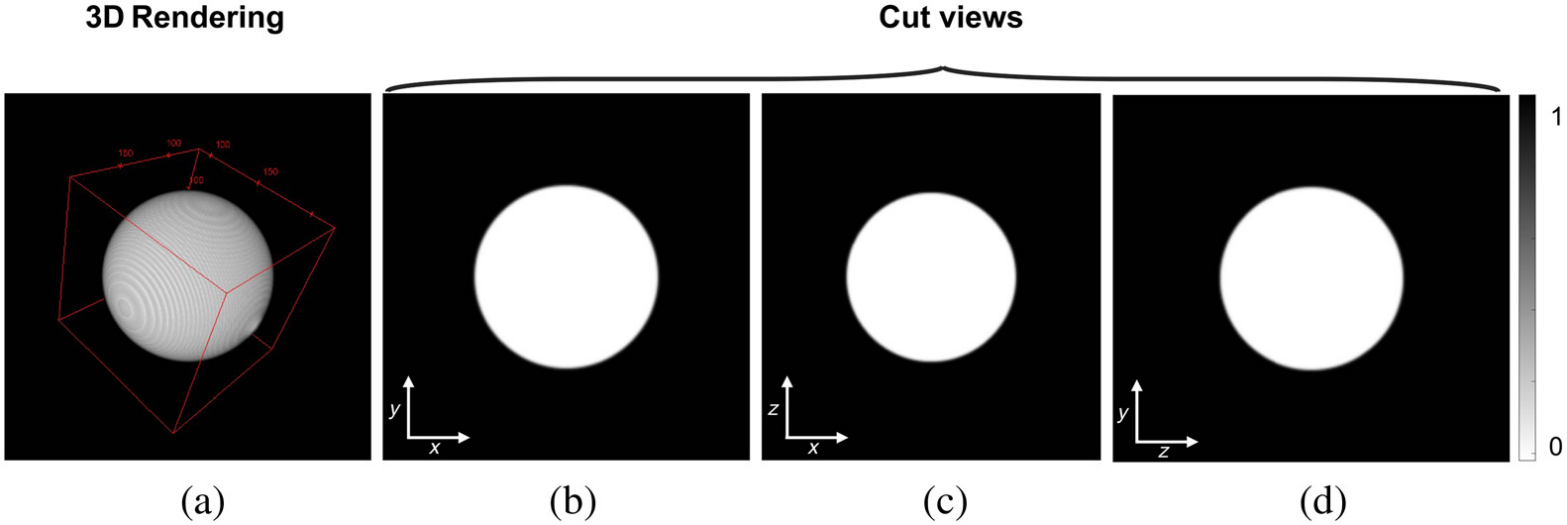
Reconstruction results of simulated PS microspheres. (a) 3D rendered result of the reconstructed phase. (b)–(d) Cut views of x–y, x–z, and y–z planes.

To assess the feasibility and efficacy of the self-supervised halo recovery method within the specified physical constraints, the light-field parameters associated with the wDPM system, as established in practice, along with simulated data generated via CytoPacq^40^ were employed for training. This process involved randomly generating a specified number of polystyrene (PS) microspheres. A benchmark known-phase synthetic dataset was constructed using a known sample refractive index and a central wavelength. In particular, based on the known spherical geometry of PS microspheres, which are typically spherical particles with diameters ranging from 3 to 5  μm, we established a thickness parameter matrix by referencing the known thickness information, assuming a uniform refractive index from 1.46 to 1.59 for the entire microsphere and a light wavelength of 574 nm. When the environmental refractive index is known, the thickness distribution can be converted into an optical phase delay using the phase matrix equation,

$$h=\frac{\lambda \Delta \phi }{2\pi (n_{s}-n_{m})},$$ (20)

where h represents the thickness of the sample, λ is the wavelength of the light source, and $n_{s}$ and $n_{m}$ are the refractive index of the sample and surrounding medium, respectively. By incorporating the random noise distribution superimposed on the microspheres, we can effectively simulate the morphological characteristics of PS and their phase distribution patterns (Fig. 5). Therefore, the synthetic phase maps were generated from known physical parameters, including sphere diameter, refractive index, and surrounding medium. These data provide a calibrated phase standard because the expected optical path length and peak phase can be analytically computed.

### 3.3 Sample Preparations

The sharing of de-identified data in this study was limited by ethical restrictions. All participants provided informed consent. This study followed the principles of the Declaration of Helsinki and was approved by the Institutional Ethics Committee of the University of Electronic Science and Technology of China (UESTC), following the protocol approved by the Institutional Ethics Committee of UESTC, Chengdu, Sichuan (Ethics Committee Protocol No. 106142022021822226).

Based on the wDPM system, we simultaneously acquired quantitative phase images of 3-μm PS microspheres (RI of 1.59) and human RBCs. First, the PS microspheres were immersed in oil (RI of 1.517) to form a suspension, which was then dispensed into the counting chamber using a pipette and covered with a coverslip. Simultaneously, human whole blood was mixed with physiological saline to prepare a blood cell suspension, which was injected into the counting chamber in the same manner as described above. The sample was placed on the motorized stage of an Olympus IX73 inverted microscope, and interference images of the sample and background were acquired at the same focal plane using a CMOS camera (DAHENG MER2-500-60U3C-L).

Fourier transform was used to perform phase reconstruction on the off-axis digital holographic images of PS microspheres, and phase unwrapping was completed using the least squares method based on the discrete cosine transform.^41^^,^^42^ For RBCs, a certain amount of human whole blood was injected into an appropriate amount of physiological saline to prepare a blood cell suspension with the desired concentration. An appropriate amount of the blood cell suspension was then aspirated using a pipette, injected into the counting chamber, and placed on the microscope stage for digital holographic imaging. Because experimentally paired halo-free RBC phase maps were unavailable, halo-free references were generated using the iterative inverse reconstruction method.

## 4 Results and Discussion

### 4.1 Experiment Results

**Fig. 6:**
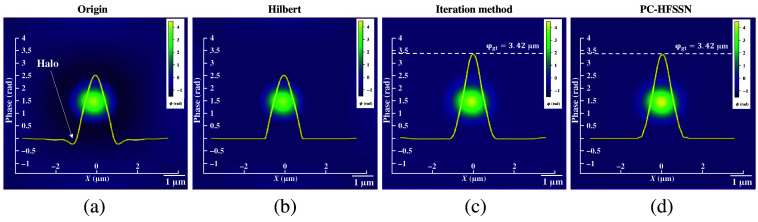
Performance evaluation of representative synthetic PS microspheres. The theoretical peak phase calculated from the sphere diameter, refractive index contrast, and wavelength is 3.42 rad, as indicated by the dashed baseline. (a) Input phase map with halo. (b) Non-iterative Hilbert transform result (edge artifact removal, sample phase height unchanged). (c) Iterative deconvolution result (edge and thickness phase synchronous correction). (d) Reconstructed results by PC-HFSSN.

To evaluate the proposed method under simulated conditions, we first generated a synthetic dataset by propagating synthesized phase samples through the wDPM forward model. Because the original phase distribution is known, this evaluation provides a theoretical reference for assessing whether each method can recover a halo-free phase. For the representative synthetic PS microsphere shown in Fig. 6, the diameter is 4  μm, the refractive index is 1.59, and the surrounding medium has a refractive index of 1.51. As shown in Fig. 6, the input with halo effects [Fig. 6(a)] exhibits pronounced negative pseudo-contours around the PS-like microsphere, and the central phase is attenuated. The non-iterative Hilbert transform method [Fig. 6(b)] reduces negative edge artifacts by means of non-negative clipping; however, it cannot fully restore the attenuated phase height. The iterative deconvolution method [Fig. 6(c)] corrects both boundary artifacts and central phase loss and matches the known numerical reference height but requires a certain number of iterations to achieve optimization. PC-HFSSN [Fig. 6(d)] achieves comparable halo suppression and peak phase recovery to the aforementioned methods through direct feedforward inference while preserving boundary distribution characteristics similar to the iterative solution. This verifies that our model has numerical consistency under a known forward model.

Next, we evaluated the simulation-to-reality performance of PC-HFSSN on 3-μm PS microspheres using experimentally acquired data that were not included in the training set. These measurements were obtained under the same wDPM configuration as that used in the forward model but included experimental perturbations, such as light source fluctuations, sensor noise, optical misalignment, and sample heterogeneity. For the measured samples, directly paired halo-free phase maps were unavailable; thus, the results of the iterative deconvolution were used as the reference solution for our deep learning approach. We further compared the reconstructed phase distributions with the theoretical phase computed based on the known diameter and refractive index difference, thereby providing an independent theoretical reference.

**Fig. 7:**
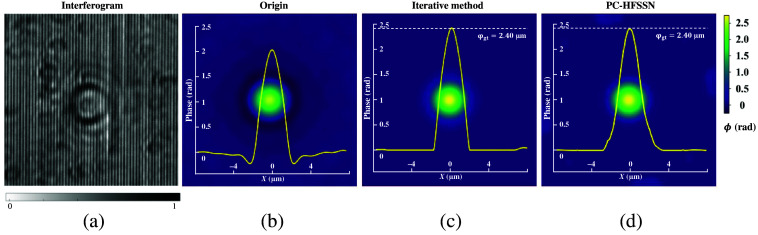
Reconstructed results of real PS microspheres. The theoretical peak phase is 2.40 rad, as indicated by the dashed baseline. (a) Digital holographic image. (b) Measured image. (c) Iteration image by L-BFGS-B. (d) Reconstructed image by PC-HFSSN.

As shown in Fig. 7, the raw measured wDPM phase contains a typical halo effect, including negative boundary contours and central phase underestimation. As shown in the line profile plotted along the microsphere diameter and the baseline of the theoretical phase peak, the results reconstructed by PC-HFSSN are closer to the theoretical reference value of the PS microsphere than the iterative results. These results indicate that PC-HFSSN not only reproduces image similarity to an iterative solver but also accurately preserves the physically realized phase magnitude of real phase objects.

We further evaluated the PC-HFSSN using the measured RBC phase acquired by the wDPM system. For RBCs, the true halo-free phase distribution cannot be directly measured from a live sample. The purpose of this comparison was to evaluate whether the PC-HFSSN could reproduce the regularized inverse solution without iterative computation and whether the recovered phase remained plausible.

**Fig. 8:**
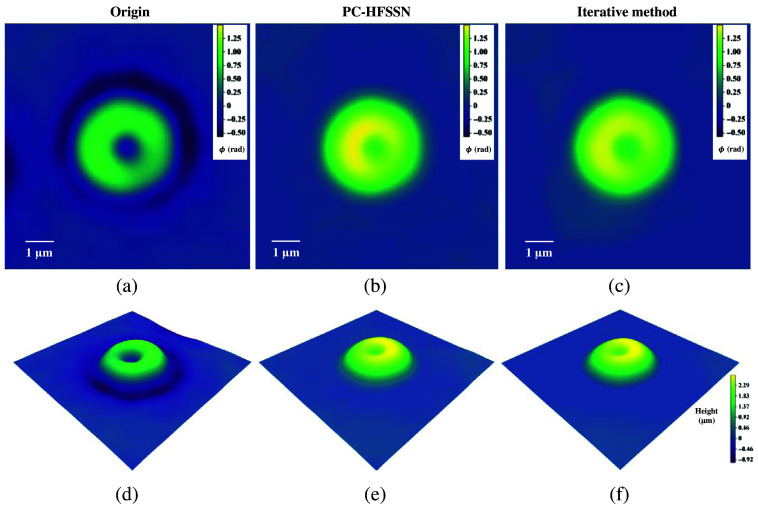
Reconstructed results of RBCs captured by the wDPM system. (a) Measured image. (b) Reconstructed image by PC-HFSSN. (c) Iteration image by L-BFGS-B. (d)–(f) Height image of the same sample in panels (a)–(c).

As shown in Fig. 8, the raw wDPM measurement exhibited phase depression and halo effect near cell boundaries [Fig. 8(a)]. PC-HFSSN effectively reduces these artifacts and recovers the biconcave morphology expected for RBCs. The thickness distribution derived from the measured phase predictions aligns with the clinically established physiological range of 2 to 3  μm.^43^ The visual and quantitative comparisons between PC-HFSSN outputs [Fig. 8(b)] and the iterative deconvolution result [Fig. 8(c)] reveal near-identical contour profiles. Furthermore, a morphological comparison of the overall three-dimensional (3D) phase distributions indicated that PC-HFSSN consistently aligned with the iterative reference results regarding both central phase recovery and boundary halo suppression.

### 4.2 Discussion

To quantify the accuracy of our self-supervised halo phase recovery method, two evaluation metrics describing numerical consistency were first introduced to reflect the effect of training the reconstruction.^44^

The first is the normalized root mean square error (NRMSE),

$$\mathrm{NRMSE}(\varphi ,\varphi_{\mathrm{ref}})=\frac{\sqrt{\frac{1}{XY}\overset{X}{\underset{i=1}{\sum }}\overset{Y}{\underset{j=1}{\sum }}{\left(\varphi (i,j)-\varphi_{\mathrm{ref}}(i,j)\right)}^{2}}}{\sqrt{\frac{1}{XY}\overset{X}{\underset{i=1}{\sum }}\overset{Y}{\underset{j=1}{\sum }}\varphi_{\mathrm{ref}}{\left(i,j\right)}^{2}}},$$ (21)

where φ(i,j) and $\varphi_{\mathrm{ref}}(i,j)$ indicate the original and reconstructed phase data, respectively.

Second, we used the structural similarity index (SSIM),

$$\mathrm{SSIM}(\varphi ,\varphi_{\mathrm{ref}})=\frac{\left(2\mu_{\varphi }\mu_{\varphi_{\mathrm{ref}}}+C_{1})(2\sigma_{\varphi ,\varphi_{\mathrm{ref}}}+C_{2}\right)}{\left({\mu_{\varphi }}^{2}+{\mu_{\varphi_{\mathrm{ref}}}}^{2}+C_{1})({\sigma_{\varphi }}^{2}+{\sigma_{\varphi_{\mathrm{ref}}}}^{2}+C_{2}\right)},$$ (22)

where μ is the mean of the data, $\sigma^{2}$ is the variance of the data, $\sigma_{\varphi ,\varphi_{\mathrm{ref}}}$ is the covariance of the two data, and c1 and c2 are two constants. The value range of SSIM is generally between −1 and 1, where 1 indicates identical images and values close to 0 indicate low structural similarity. The larger the value, the more similar are these two images.

**Table 1:** Reconstructed results of measured RBCs based on the non-iterative Hilbert method, HFDNN, and PC-HFSSN.

Sample	Methods	NRMSE	SSIM
PS	Hilbert^11^	0.0134	0.7787
	HFDNN^17^	0.0073	0.7985
	**PC-HFSSN**	**0.0062**	**0.8067**
RBCs	Hilbert^11^	0.0950	0.8296
	HFDNN^17^	0.0189	0.9125
	**PC-HFSSN**	**0.0076**	**0.9459**

As shown in Table 1, for PS microspheres, which present relatively simple and symmetric morphologies, the model achieved an NRMSE of 0.0062 and an SSIM of 0.8067. In contrast, biological specimens, such as RBCs, exhibit significant structural heterogeneity, characterized by irregular contours and varying phase gradients. Despite this complexity, our network maintained exceptionally high fidelity. We also observed that the relatively lower SSIM for PS should be interpreted considering the mathematical nature of SSIM. SSIM is computed from local luminance, contrast, and structural terms, and its structural component depends strongly on local variance and covariance. Smooth spherical phase objects contain large, slowly varying regions with weak local texture; therefore, small low-frequency deviations in phase height or boundary position can reduce SSIM, even when the absolute phase error is small. In contrast, RBCs contain a more heterogeneous morphology, including biconcave profiles, sharper boundaries, and spatially varying phase gradients. PC-HFSSN achieved an NRMSE of 0.0076 and an SSIM of 0.9459 relative to the iterative reference. These features provide stronger local structural anchors, which can naturally yield a higher SSIM when the reconstructed and reference images share a similar morphology. Compared with the non-iterative Hilbert method and HFDNN, PC-HFSSN shows improved consistency with the iterative reference while maintaining efficient feed-forward inference.

This performance consistency is firmly underpinned by the invariance of the OTF between the simulated training environment and the physical wDPM system. Because identical forward propagation physics governs both domains, the PC-HFSSN framework can seamlessly generalize to diverse biological phase data acquired by the current optical setup. Consequently, the model successfully processes morphologically complex biological specimens without requiring sample-specific network retraining or new paired experimental datasets.

To further evaluate the halo-removal performance of PC-HFSSN on real samples, we measured the dry mass and surface area of RBCs as complementary quantitative metrics to assess the influence of halo correction on relevant cellular physical properties. The dry mass ρ of RBC can be obtained from the height map h(x,y) as

$$\rho =\frac{1}{\alpha }\iint_{D}h(x,y),$$ (23)

where D represents the area of a single cell. The specific refractive increment α describes the proportional relationship between biomolecular mass concentration and refractive index variation. For RBCs, whose intracellular content is mainly composed of hemoglobin, α is set to $0.19\;\mu m^{3}/\mathrm{pg}$ in our study.^45^ The surface area of RBC is determined using Monge parameterization, where the contribution of each pixel can be calculated as^46^

$$dA=\sqrt{1+h_{x}^{2}+h_{y}^{2}}dx\;dy,$$ (24)

where dx and dy denote the physical pixel sizes along the x and y directions, respectively, and $h_{x}$ and $h_{y}$ represent the height gradients along the corresponding directions. The surface area of each cell is calculated by summing the area elements over the cell region, with the projected area taken into account because the cells are assumed to lie flat on the coverslip.

**Fig. 9:**
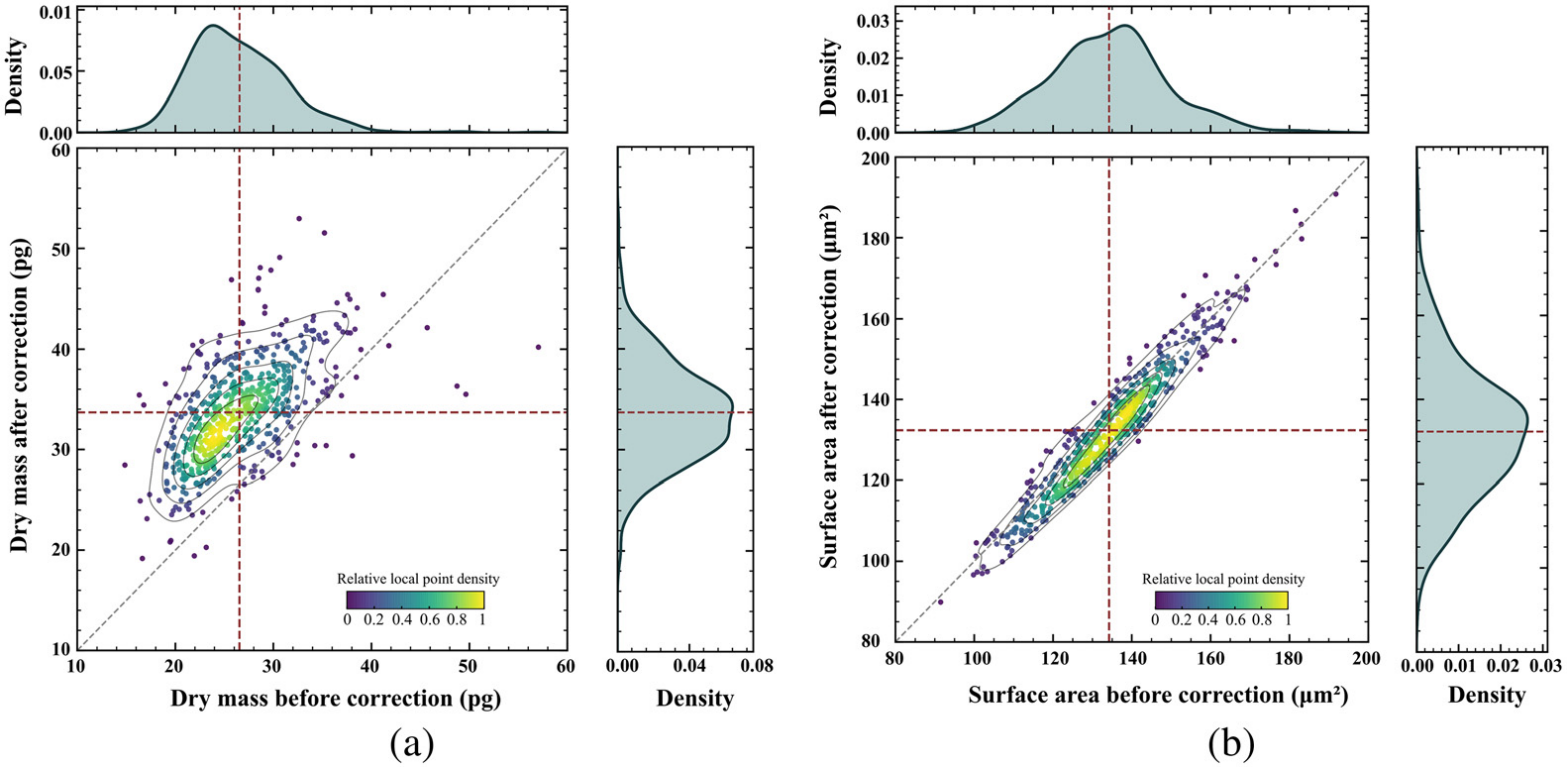
Density distribution and scatter plots of (a) dry mass and (b) surface area before and after halo correction. The red dotted lines are set at the position of the mean values.

**Table 2:** Mean and standard deviation (SD) of RBC dry mass and surface area before and after halo correction.

Variables	With halo correction	Mean	SD
Dry mass (pg)	√	33.7	4.9
	×	26.5	5.0
Surface area (${\mu m}^{2}$)	√	132.4	15.5
	×	134.2	14.8

Blood samples were collected from healthy patients and diluted with physiological saline. At room temperature, digital holograms of 590 RBCs were acquired using the wDPM system for subsequent analysis. We quantified the distributions of dry mass and surface area before and after halo correction, and visualized the results using density distribution and scatter plots, as shown in Fig. 9. The detailed statistical results are summarized in Table 2. It can be observed that the halo correction by PC-HFSSN had markedly different effects on RBC dry mass and surface area. For dry mass, the estimated value for the same RBC is generally higher after halo correction than before correction. The mean dry mass increased from 26.5 to 33.7 pg after correction. This result indicates that the halo artifact leads to an underestimation of the integrated RBC phase and the corresponding dry mass, whereas PC-HFSSN effectively compensates for this underestimation and yields dry mass estimates closer to the reasonable range.^47^ In contrast, the RBC surface area shows only minor changes after halo correction. The scatter points of RBC surface area are tightly distributed along the diagonal reference line, and the density contours exhibited a narrow, elongated linear pattern, indicating high consistency between the RBC surface area measurements before and after correction. These results suggest that the halo correction mainly compensates for phase underestimation caused by halo artifacts while having limited influence on the surface area determined by the cellular geometric outline. Therefore, the correction process does not substantially alter the morphology or size distribution of RBCs.

In addition to reconstruction accuracy and morphological consistency, computational efficiency is also an important consideration for QPI applications. Therefore, we compared the computational efficiency of PC-HFSSN with that of the iterative L-BFGS-B reconstruction method. Both methods were tested on the same hardware with the same phase images. The execution time or time consumption was calculated as the average time required for inference or reconstruction per image across several trials. For PC-HFSSN, the reported time corresponded to a single forward pass. In contrast, the time for L-BFGS-B included all iterations required to reach convergence using the same stopping criteria as in the experiments. In addition, we evaluated the computational complexity by analyzing the floating point operations (FLOPs). For PC-HFSSN, FLOPs were calculated based on the convolutional layers and sampling operations of the U-Net backbone. For the iterative L-BFGS-B solver, the computational cost was estimated by timing one objective function or gradient evaluation and extrapolating it using the total number of function evaluations.

Currently, each L-BFGS-B evaluation contains two large complex convolutions with a 256×256 kernel, requiring ∼68.7 GFLOPs and 94.3 ms per evaluation. To reach convergence, iterative deconvolution algorithms typically require 25 to 50 evaluations, which corresponds to 1.72 to 3.44 Tera-FLOPs and 2.51 to 5.02 s per image. We also considered the situation in which the convolutions were replaced by fast Fourier transform-based convolutions; the estimated arithmetic cost decreased to ∼2.86 Giga-FLOPs (GFLOPs) per evaluation. However, because the L-BFGS-B reconstruction still requires repeated sequential gradient evaluations before convergence is reached, the estimated runtime remains 0.53 to 1.05 s per image. In contrast, PC-HFSSN requires only one feed-forward pass, with 6.12 GFLOPs and 3.74 ms per image. Thus, the main computational advantage of PC-HFSSN is the replacement of repeated iterative optimizations with fixed-cost parallel inference.

## 5 Conclusion

In summary, we report a PC-HFSSN that couples angular spectrum diffraction theory with a NA-based Gaussian illumination approximation to efficiently eliminate the halo effect in wDPM. By embedding a differentiable forward propagation model directly into the loss function, the network enforces strict adherence to optical physical laws while capturing data-driven features. This self-supervised approach, utilizing a lightweight U-Net, effectively circumvents the challenge of obtaining ground truth for dynamic biological samples. Experimental validation confirmed that PC-HFSSN matched the accuracy of regularized iterative algorithms but obviated complex iterative procedures, thereby significantly boosting computational efficiency. Furthermore, our multi-scale simulation training strategy facilitated robust generalization to unlabeled real biological samples, successfully bridging the simulation-to-reality gap. Although the current study primarily validated the PC-HFSSN framework using foundational models, such as PS microspheres and RBCs, the inherent flexibility of our physics-constrained approach allows for broader applications. Future investigations will expand the synthetic training datasets to encompass more morphologically complex biological specimens, such as other complex cell cultures and organoids, as well as extensions into multi-wavelength systems. Ultimately, this framework establishes a new paradigm for high-fidelity quantitative phase imaging at the intersection of computational optics and biomedicine.

## Data Availability

The custom software code underlying the proposed PC-HFSSN framework is available on GitHub and can be accessed at https://github.com/moemilqpi/Halo-Free-SSN. The datasets generated and/or analyzed during the current study are not publicly available owing to their large file sizes and ongoing subsequent research but are available from the corresponding author upon reasonable request.
